# Kinesiophobia Levels in Patients with Multiple Sclerosis: A Case-Control Investigation

**DOI:** 10.3390/biology11101428

**Published:** 2022-09-29

**Authors:** Francisco Javier Ruiz-Sánchez, Maria do Rosário Martins, Salete Soares, Carlos Romero-Morales, Daniel López-López, Juan Gómez-Salgado, Ana María Jiménez-Cebrián

**Affiliations:** 1 Health and Podiatry Group, Department of Health Sciences, Faculty of Nursing and Podiatry, Industrial Campus of Ferrol, Universidade da Coruña, 15403 Ferrol, Spain; 2 Instituto Politécnico de Viana do Castelo, Escola Superior de Saúde, Rua D. Moisés Alves Pinho 190, 4900-314 Viana do Castelo, Portugal; 3 Faculty of Sport Sciences, Universidad Europea de Madrid, Villaviciosa de Odón, 28670 Madrid, Spain; 4 Department of Sociology, Social Work and Public Health, Faculty of Labour Sciences, University of Huelva, 21007 Huelva, Spain; 5 Safety and Health Postgraduate Programme, Universidad Espíritu Santo, Guayaquil 092301, Ecuador; 6 Instituto de Investigación Biomédica de Málaga (IBIMA), Nursing and Podiatry Department, Faculty of Health Sciences, University of Malaga, 29071 Malaga, Spain

**Keywords:** multiple sclerosis, foot health, kinesiophobia, fear, movement

## Abstract

**Simple Summary:**

Fear of movement or kinesiophobia is a health problem related to an irrational fear of physical movement and fatigue that can lead to a limitation of functional capacity and a decrease in daily physical activity. Thus, the purpose of this study was to test the level of kinesiophobia in people with multiple sclerosis and compare it with a group of healthy people. The findings of this research support evidence that kinesiophobia is higher in people with multiple sclerosis than in a healthy control group. Therefore, people with multiple sclerosis should be evaluated and monitored to plan the treatment and preventive care activities of a multidisciplinary health team (podiatrists, physiotherapists, nurses, physicians, occupational therapists) in the attempt to improve their quality of life, autonomy, and wellbeing.

**Abstract:**

Fear of movement or kinesiophobia is an irrational fear of physical movement and fatigue that causes a limitation of functional capacity and decreased physical activity. The purpose of this study was to ascertain the level of kinesiophobia in people with multiple sclerosis (pwMS) and compare it with a group of healthy people, through the Tampa Scale for Kinesiophobia with 11 items (TSK-11). Method: A total of 116 subjects were recruited in a multicenter case-control study; 58 subjects suffered from MS and 58 were healthy subjects from different associations and the same locality. To assess the levels of fear of movement, the Spanish version of the TSK-11 self-questionnaire was used. Results: Most pwMS suffer from some degree of kinesiophobia (TSK-11 ≥ 18), and 60.3% had moderate to maximum kinesiophobia scores (TSK-11 ≥ 25). In contrast, healthy subjects presented a percentage of kinesiophobia from none to moderate (82.7%). Conclusions: Kinesiophobia is higher in pwMS than in the healthy control group. Accordingly, individuals showing pwMS should be assessed and monitored in order to diagnosed initial kinesiophobia levels, to allow planning treatment and preventive care activities that may improve the foot health and overall health in this group of patients.

## 1. Introduction

Fear of movement or kinesiophobia is an excessive, irrational, and debilitating fear of performing a physical movement, due to a feeling of vulnerability to a painful injury or a new injury [1,2]. It is a fear of the physiological symptoms of fatigue or exhaustion or, even more broadly, fear of physical or mental discomfort. Thus, several defense mechanisms may appear, such as: repression (conscious inhibition), negation (there is no need for movement), simulation and projection (people already initiated to sport postpone their sports activity), or rationalization (lack of time) [3]. The prevalence of kinesiophobia varies according to the study population, being 66.6% in patients with lupus erythematosus [4], 85.7% in patients with knee osteoarthritis [5], 76.3% in patients with coronary artery disease [6,7], 69.2% in patients before reconstruction of the anterior cruciate ligament of the knee [8], and 62.2% in patients after hip arthroplasty [9].

Multiple sclerosis (MS) is a demyelinating and chronic inflammatory illness of autoimmune etiology. The main factors associated with MS development involve genetic, environmental, and exogenous agents [10]. It is estimated that 2.8 million people suffer from this disease [11], but according to data from the World Atlas of MS, the number is higher, since there are people who live with the disease but in their country do not have access to a diagnosis due to a lack of qualified medical professionals or because of the cost associated with diagnosis [12]. MS is accompanied by the presence of symptoms arising from injuries to the spinal cord and the optic nerves or brain that can influence the postural balance, as well as walking problems, muscle weakness, spasticity, fatigue, and higher fall risk [13].

Continuous physical activity of the musculoskeletal system has a positive impact on MS according to a recent study, which shows that physical activity can help reduce fear and improve confidence in movement. However, physical activity recommendations must be adapted to the individual’s abilities so that they are feasible, thus giving a sense of achievement and increasing motivation [14].

It is essential to understand the factors that influence the pain of pwMS, since there may be an association of pain with kinesiophobia, this being clinically useful for the development of clear interventions in patients with chronic pain [15]. In pwMS, fear of movement is an obstacle for continuous physical activity as well as for normal development of daily habits, impacting quality of life and biopsychosocial well-being [16]. It is necessary to identify kinesiophobia in this group of patients to prevent a sedentary lifestyle and disease progression, thus improving therapeutic care as pwMS spend a significant amount of sedentary time with a higher disability status [17]. The Tampa Scale for Kinesiophobia (TSK-11) has been used to measure kinesiophobia levels [18].

After a search of the published literature, no study was found on this psychological factor (kinesiophobia) in pwMS compared to healthy subjects of the same age, sex, and BMI; therefore, the present study was conducted by administering the TSK-11 to a group of pwMS and to a matched group of healthy individuals, in order to assess kinesiophobia levels.

## 2. Materials and Methods

### 2.1. Design and Sample

An observational, analytical, and multicenter case-control study was carried out in which subjects from MS associations in Granada (*n* = 8), Campillos, Malaga (*n* = 8), Francisco Ruiz Sánchez Podiatry Clinic, Cadiz (*n* = 4), and the Regional Hospital of Ronda (*n* = 38) were recruited.

The study population consisted of pwMS (*n* = 58) and subjects without MS (*n* = 58). The patients were recruited using a convenience sampling method.

PwMS were included after being informed by the MS association that a study of the foot and fear of movement was to be carried out. The control subjects were recruited from among healthy people from an ambulatory health care center in the same locality.

The inclusion criteria were subjects between 18–66 years old, of both sexes, able to walk, and who authorized their participation with the signing of a consent form.

The exclusion criteria were to have another neurodegenerative disease other than MS, persons with immunosuppression, persons with experienced previous trauma and feet surgery records, lack of or partial autonomy in daily activities, as well as those who refused to sign the consent form or were incapable of understanding the instructions necessary to carry out the present study. Cases and controls were matched for age, gender, and BMI.

### 2.2. Sample Size Calculation

The Epidat 4.2 program (Consellería de Sanidade, Xunta de Galicia, España; Organización Panamericana de la salud (OPS-OMS); Universidad CES, Colombia) was used to calculate the sample size, obtaining specific levels of confidence, power, and groups of equal size. A sample of 102 participants (51 per group) was obtained with a confidence level of 70%, an odds ratio of 2.0, a power of 0.80 and an exposure of 66.67% for the MS group, and 50% in the control group. Finally, for operational and safety reasons, a total of 58 patients (in each group) were used in this study.

### 2.3. Procedure

At the beginning of the data collection, the research podiatrist asked the participant a series of questions in order to obtain their general state of health. The first data collected were anthropometric variables such as age, sex, height, weight, and body mass index, as well as the years of evolution and type of MS. The researcher performed a physical examination of the participant’s foot where different pathologies were selected, such as onychocryptosis, drop foot, pes cavus or flat feet, dermatomycosis, hyperhidrosis, and hallux abductus valgus. Using a visual analog scale, the patient had to indicate from 0 (no pain) to 10 (a lot of pain) the level of pain in the foot: forefoot, midfoot, and hindfoot; since a scale from 0 to 10 was used in our study, it was established that pain would be classified from 1 to 3 as mild pain, 4 to 7 as moderate pain, and from 8 to 10 as severe pain [19]. Finally, the Tampa Scale for Kinesiophobia (TSK-11), Spanish version, was administered. [20]. It is a psychometric questionnaire for diagnosis, prognosis, follow-up, and clinical orientation, used to assess a patient’s fear of suffering a new injury due to movement [21]. It is a Likert-type scale that goes from 1 (totally disagree) to 4 (totally agree) where the subjects indicate their degree of agreement with each statement. This 11-item version provides the best fit encompassing these two factors, as well as being a variant for pain diagnoses. TSK-11 scores ranged from 11 to 44, with the higher the score, the greater the fear of pain with movement. The total scores were classified as follows: 11–17 points, without fear of movement; 18–24 points, slight fear of movement; 25–31 points, moderate fear of movement; 32–38 points, severe fear of movement; 39–44, maximum fear of movement [22,23].

This scale was completed by Gomez-Perez et al. in terms of validity and reliability, which together with an internal consistency of 0.78 with Cronbach and a test-retest with an ICC of 0.82 show this scale to have adequate psychometric properties.

### 2.4. Ethics Procedure

A favorable assessment was obtained from the Ethics Committee of the University of Malaga (CEUMA) with registration number 32-2021-H and the Ethics Committee of the Biomedical Research Institute of Malaga with code 5002V01. In addition, the subjects gave their permission to participate in the study by signing the informed consent form. The study was developed following the ethical principles of clinical research in human beings [24,25].

### 2.5. Statistics

We used the Kolmogorov–Smirnov test to evaluate the normality of the sample. Between-group comparisons were made using the Chi-square test to test categorical variables and the Student t-test for independent samples for continuous variables. In all analyses, a *p* value of less than 0.05 was considered statistically significant.

## 3. Results

### 3.1. Descriptive Data

A total population of 116 subjects was included in the study and no participants were excluded. Thus, they were divided into pwMS (*n* = 58, for the case group) and subjects without MS (*n* = 58, for the control group). Both groups were matched for age, sex, and BMI.

All data showed a normal distribution (*p* > 0.05) between groups for descriptive data. These data are shown in Table 1.

### 3.2. Outcome Measurements

Table 2 shows that, in general, the pwMS presented higher scores in TSK-11 than the healthy subjects. In addition, 93.1% of participants with MS had the relapsing-remitting type (*n* = 54), 3.4% primary progressive (*n* = 2) and 3.4% secondary progressive (*n* = 2) types. It can be seen that in this study there is a greater presence of women (70.7%) with MS. Most of the pwMS did not present foot pain (67.2%), and regarding identification of percentages of pain in the three regions of the foot, mild pain was the most predominant: forefoot (62.1%), midfoot (72.4%), and hindfoot. (77.6%).

In Table 2, it should be noted that the majority of pwMS had some degree of kinesiophobia (TSK-11 ≥ 18), and 65.9% had moderate to maximum kinesiophobia scores (TSK-11 ≥ 25). In contrast, healthy subjects presented a percentage of kinesiophobia from none to slight (82.7%).

## 4. Discussion

The objective of our study was to analyze the level of kinesiophobia in pwMS and to compare them with healthy subjects. Our theory was that pwMS suffer more frequently from kinesiophobia than healthy controls.

The kinesiophobia scores of 58 subjects with MS and 58 healthy subjects were compared. As a result, it was found that the majority of MS patients suffered from kinesiophobia at one of the four levels (slight, moderate, severe, and maximum). The scores indicate that 34% of the pwMS suffered from a low level of kinesiophobia, and 65.6% presented high levels of kinesiophobia (scores TSK-11 ≥ 25).

In this way, our results are in the line with a previous research Wasiuk-Zowada et al., that relates the increase of the probability of occurrence or exacerbating of kinesiophobia in people with neurological illnesses such as stroke patients, or patients suffering from multiple sclerosis or Parkinson’s disease [26].

Furthermore, in the research of Baglan et al. it was found that in patients with lupus erythematosus and kinesiophobia, 66% is associated with depression and pain [4]. Other studies, such as that of patients with chronic knee osteoarthritis, present a high level of kinesiophobia (85%), which should be considered as a risk factor for chronic pain, physical inactivity, disability, and poor quality of life [5]. From the results obtained in our study we can see that most of the pwMS presented mild pain for the three regions of the foot, where there are significant differences between pain and kinesiophobia (*p* < 0.05, Chi-square test were used).

In another risk group, such as elderly people with coronary artery disease, kinesiophobia is a severe problem (76%), this being a risk factor for the progression of this disease.

Currently, only one previous study was found demonstrating levels of kinesiophobia in pwMS. It classifies them according to the Extended Disability Status Scale (EDSS) into three groups from least to greatest disability; thus, the first group with the least disability is the one with the least fear of movement (19% of the cases), and the second and third group, with the highest degree of disability, present higher percentages of kinesiophobia (74% of the cases) [26].

However, the study scores are based on the 17-item version (TSK-17) [27], not being comparable with the results of our study that used the TSK-11. In addition, this study does not make a comparison in terms of healthy subjects.

As a value to be scored, the level of kinesiophobia in MS has a negative influence on the subjects who suffer from it, and this is confirmed by Wasiuk-Zowada et al., that the problem of kinesiophobia is significant in patients with MS, causing a low level of physical activity [28]. Similarly, it occurs in patients with chronic musculoskeletal pain, who present a high level of kinesiophobia, being higher in adult men (47.5%) [29].

Likewise, even in those with walking daily, MS may have a significant effect on gait and motor impairment and cause alterations in gait, muscle deficits, and loss of balance, thus increasing the risk of falls leading to kinesiophobia [30,31].

More studies are needed to find out if gait and balance alterations in subjects with MS favor fear of movement, as well as to investigate the different interventions that could reduce this fear.

Finally, this study is important because it focuses on evaluating and classifying kinesiophobia in pwMS compared to a control group of healthy subjects. This research presents some limitations, such as: a larger and more diverse (people from other countries) sample size would be beneficial to expand the strength of this investigation and help to recognize if there is a culture where this relation does not occur and detect the mechanisms involved. Thus, future investigations would benefit from increased sample sizes, and research across various ethnicities, cultures, and other living locations.

## 5. Conclusions

Kinesiophobia is higher in pwMS than in the healthy control group. Accordingly, individuals showing pwMS should be assessed and monitored in order to diagnose initial kinesiophobia levels, to allow planning treatment and preventive care activities that may improve the foot health and the overall health in this group of patients.

## Figures and Tables

**Table 1:** Descriptive and socio-demographic data of the sample.

Demographic and Descriptive Data		Total Group *n* = 116 Mean ± SD	MS *n* = 58 Mean ± SD	Healthy *n* = 58 Mean ± SD	*p* Value
Age (Years)		47.38 ± 10.62 (24–66)	47.38 ± 10.68 (24–66)	47.38 ± 10.65 (24–66)	1.000 †
Weight (kg)		71.36 ± 12.75 (46–105)	70.19 ± 13.31 (46–105)	72.53 ± 12.16 (47–100)	0.324 †
Height (cm)		167.68 ± 8.43 (150–188)	167.24 ± 8.49 (150–183)	168.12 ± 8.43 (154–188)	0.577 †
BMI (kg/m^2^)		25.32 ± 4.14 (18.0–37.5)	24.98 ± 3.94 (18.0–37.5)	25.65 ± 4.34 (18.4–37.2)	0.391 †
Time since MS diagnosis (years)		N/A	12.55 ± 8.53 (1–33)	N/A	N/A
Sex (%)	Male Female	34 (29.3%) 82 (70.7%)	17 (29.3%) 41 (70.7%)	17 (29.3%) 41 (70.7%)	1.000 ‡

Abbreviations: BMI: body mass index; N/A, not applicable; † Student’s *t*-test was applied for independent samples. ‡ The Chi-square test was used. In all analyses, *p* < 0.05 (with a 95% confidence interval) was considered statistically significant.

**Table 2:** Comparisons of TSK-11 scores and categories between pwMS and without pwMS.

Outcome Measurements			Total Group (*n* = 116)	MS Mean ± SD (*n* = 58)	Healthy Mean ± SD (*n* = 58)	*p*-Value (Cases vs. Controls)
TSK-11 Category	Fear of movement	No	45 (38.8%)	11 (19%)	34 (58.6%)	**<0.001**
		Slight	23 (19.8%)	9 (15%)	14 (24.1%)	
		Moderate	28 (24.1%)	20 (34.5%)	8 (13.8%)	
		Severe	12 (10.3%)	11 (19%)	1 (1.7%)	
		Maximum	8 (6.9%)	7 (12.1%)	1 (1.7%)	
TSK scores			N/A	27.72 ± 8.66 (12–44)	17.78 ± 6.58 (11–40)	**<0.001 †**

Abbreviations: BMI: body mass index; TSK-11, Tampa Scale Kinesiophobia; N/A, not applicable. Frequency, percentage (%) and Chi-squared tests (χ2) were utilized. † Student’s *t*-test was applied for independent samples. In all the analyses, *p* < 0.05 (with a 95% confidence interval) was considered statistically significant (bold).

## Data Availability

The dataset supporting the conclusions of this article is available upon request to f.ruiz@udc.es in the Research, Health and Podiatry Group, Department of Health Sciences, Faculty of Nursing and Podiatry. Industrial Campus of Ferrol, Universidade da Coruña, 15403, Ferrol; Spain.
